# Sequential Photodynamic Therapy with Phthalocyanine Encapsulated Chitosan-Tripolyphosphate Nanoparticles and Flucytosine Treatment against *Candida tropicalis*

**DOI:** 10.3390/pharmaceutics11010016

**Published:** 2019-01-04

**Authors:** Yi-Hsuan Hsieh, Wen-Ching Chuang, Kun-Hua Yu, Cheng-Ping Jheng, Cheng-I Lee

**Affiliations:** 1 Department of Clinical Pathology, Dalin Tzu Chi Hospital, Buddhist Tzu Chi Medical Foundation, Chia-Yi 62247, Taiwan; dm989587@tzuchi.com.tw; 2 Department of Biomedical Sciences, National Chung Cheng University, Min-Hsiung Chia-Yi 62102, Taiwan; danny7910791@gmail.com (W.-C.C.); ykhuna@gmail.com (K.-H.Y.); chengpingkevincheng@hotmail.com (C.-P.J.); 3 Center for Innovative Research on Aging Society (CIRAS), National Chung Cheng University, Min-Hsiung Chia-Yi 62102, Taiwan; 4 Center for Nano Bio-detections, Advanced Institute of Manufacturing with High-tech Innovations (AIM-HI), National Chung Cheng University, Min-Hsiung Chia-Yi 62102, Taiwan

**Keywords:** photodynamic therapy, *Candida tropicalis*, chitosan, phthalocianine, flucytosine

## Abstract

Antibiotic resistance has become a crisis. *Candida tropicalis* (*C. tropicalis*) is one of the most highly virulent and drug-resistant pathogens. An alternative antimicrobial therapy to eradicate *C. tropicalis* effectively, without the risk of developing drug-resistance, is needed. Photodynamic therapy (PDT) is an alternative therapy that does not carry the risk of undesired drug resistance. To target the pathogens and to enhance the cellular penetration of the applied photosensitizer, we fabricated cationic chitosan/tripolyphosphate nanoparticles to encapsulate phthalocyanine. Our strategy promotes the uptake of phthalocyanine four-fold. This enhanced PDT can effectively inhibit planktonic *C. tropicalis*, such that only ~20% of *C. tropicalis* in the test survived; but it has a limited ability to inhibit adherent *C. tropicalis*. Further tests with adherent *C. tropicalis* indicated that sequential treatment with PDT and flucytosine significantly eliminates pseudohyphae and yeast-like *C. tropicalis* cells. The cell viability is only ~10% after this sequential treatment. This study provides evidence of an effective therapy against drug resistant *C. tropicalis*, and this strategy can be potentially applied to other pathogens.

## 1. Introduction

Antibiotic resistance has become a crisis [1]. Candidiasis is one of the most frequent fungal infections [2]. In the intensive care unit of medical centers, infections with *Candida* species (*Candida* spp.) have become very common [3]. Candidiasis causes high mortality in patients with immunodeficiency [4]. *Candida albicans* (*C. albicans*) is the most commonly encountered human fungal pathogen [5]. In recent years, the overuse of antifungal drugs has led to resistance in many non-*albicans Candida* spp., such as *Candida tropicalis* (*C. tropicalis*) and *Candida glabrata* (*C. glabrata*) [6,7]. In addition, the resistance of these species to azole drugs results in serious infections [8,9]. *C. tropicalis* is an extremely common pathogen that causes human disease in tropical countries [10]. The virulence of *C. tropicalis* can be more severe than that of *C. albicans* in the human intestine, particularly in oncology patients [11,12]. The incidence of urinary tract infections with *C. tropicalis* is second to that with *C. albicans* in some Asian countries [13,14].

In the treatment of candidiasis, the drugs that are commonly used to treat *Candida* infections include amphotericin B, fluconazole, liposomal amphotericin B, and flucytosine [15,16]. The antifungal mechanisms of these antifungal agents are different [17]. Amphotericin B interacts with ergosterol to disrupt fungal cell membrane by pore formation. Fluconazole destabilizes the membrane structure through reducing the synthesis of ergosterol and incorporating the precursor of sterol into the cell membrane. Flucytosine interferes with the synthesis of fungal proteins and DNA. All these antifungal agents have some side effects [17]. With the use of these chemical antifungal agents, the recurrence of infection after treatment is very common [18], mainly due to the development of resistance [19,20]. Similar to *C. albicans*, *C. tropicalis* is a polymorphic fungus that can grow either as ovoid-shaped budding yeast or as parallel-walled true hyphae. As elongated ellipsoid cells, pseudohyphae develop during the transition between budding and hyphal growth, and this transition is essential for virulence [21]. Biofilms consist of pseudohyphae, and hyphae are notorious for intrinsic resistance to conventional antifungal therapeutics [8].

Photodynamic therapy is a promising alternative treatment in cancers [22] and microbial diseases [23,24]. The combination of chemical treatment and photodynamic therapy (PDT) greatly enhances antimicrobial efficiency in *C. albicans* [25]. Many photosensitizers have been approved by the Food and Drug Administration in the US, including Photofrin, Levulan, Metvix, and Visudyne [26]. Compared with traditional therapies involving antimicrobial medicines, PDT has the advantages of localized application, low risk of side effects, and nonexistent drug-resistance [27]. However, the use of PDT in microbial infections is limited in comparison to its use for cancer treatment. Considering the significant increase of drug-resistant *Candida* infections, it is important to develop antimicrobial PDT as an alternative therapy to eradicate pathogenic microorganisms effectively, without risking drug-resistance.

Photodynamic therapy is a form of phototherapy involving three main components—light, a photosensitizer, and oxygen molecules. The photosensitizer is excited from the ground singlet state to the excited singlet state when it receives light energy at a specific wavelength. The photosensitizer in the excited singlet state crosses to the first triplet state. When returning to the ground state, the photosensitizer releases some energy to a nearby triplet molecule oxygen (^3^O_2_), forming the excited state singlet oxygen (^1^O_2_) in a type II reaction. In the type I reaction, the excited photosensitizer reacts with the biomolecule and transfers the energy through electron transfer, producing free radicals. Further reaction between singlet oxygen and free radicals leads to the formation of a reactive oxygen species. The photosensitizer is a critical mediator in PDT. However, the solubility of photosensitizers is usually low. Furthermore, tumor-targeted forms of PDT have been developed [28,29]. The development of PDT targeted to pathogens is in progress [30], but it is not as mature yet as tumor-targeted PDT. The poor solubility and selectivity limit the applications of PDT. Nanotechnology has been applied to PDT to overcome the poor water-solubility of photosensitizers and to enhance their efficiency [31]. Encapsulation of hydrophobic photosensitizers with hydrophilic carriers increases the efficiency of drug delivery and systemic administration. Chitosan, a polysaccharide produced from the deacetylation of chitin, has been widely used as a delivery particle due to its desirable properties, which include biocompatibility, biodegradability, and lack of toxicity [32]. Therefore, chitosan-based materials have been used in PDT [33,34,35]. Fabrication of chitosan-based nanoparticles can be carried out by an intermolecular ionic cross-linking between protonated chitosan and anionic phosphate groups, such as tripolyphosphate (TPP) [34,36]. In addition, chitosan/TPP nanoparticles carrying the desired charge can be fabricated by adjusting the ratio of the chitosan and TPP.

Phthalocyanine derivatives, which have similar structures to porphyrin, have been used as photosensitizers [26,37]. Similar to the situation with other photosensitizers, their low solubility and poor cell penetrability limits their application to PDT. To overcome these limitations, we used a nanotechnology approach, in which chitosan/TPP nanoparticles (NP) were the carrier of phthalocyanine to develop an efficient antimicrobial PDT against *C. tropicalis*. The physical, chemical, and cellular properties of phthalocyanine encapsulated chitosan/TPP nanoparticles (FNP) were characterized. The antimicrobial effect of PDT using FNP was evaluated in both planktonic and adherent cultures of *C. tropicalis*. Antimicrobial activity in the combination treatment of FNP-PDT and flucytosine against *C. tropicalis* was evaluated.

## 2. Materials and Methods

### 2.1. Preparation of Chitosan and Tripolyphosphate

Chitosan (Sigma-Aldrich, St. Louis, MO, US) was added to 100 mL of acetic acid at 2% (*v*/*v*) to reach a concentration of 2% (*w*/*v*), and stirred at room temperature overnight. After the chitosan was completely dissolved, the pH of the solution was adjusted to 6, and the solution was then filtered with a 0.22 μm membrane. Sodium tripolyphosphate (Sigma-Aldrich, St. Louis, MO, US) was dissolved in H_2_O to reach a concentration of 1% (*w*/*v*), and then filtered with a 0.22 μm membrane. In the subsequent experiment, the chitosan and TPP solutions were diluted with H_2_O to the desired concentration.

### 2.2. Fabrication of FNP

**Figure 1:**
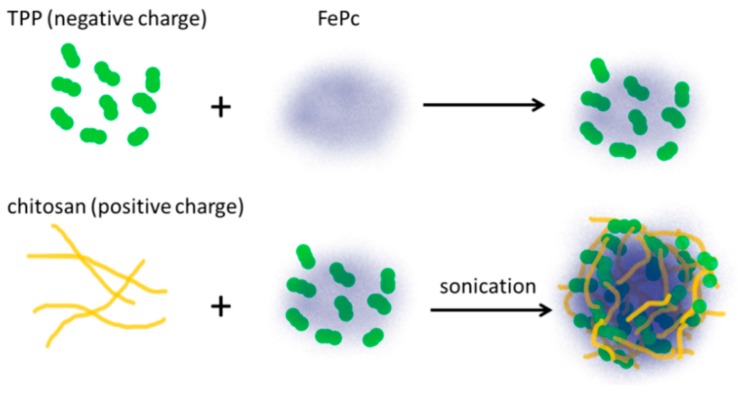
A schematic diagram for the fabrication of phthalocyanine encapsulated chitosan/TPP nanoparticles (FNP) from chitosan, tripolyphosphate (TPP), and phthalocyanine-4,4′,4″,4‴-tetrasulfonic acid (FePC).

To generate FNP, the photosensitizer was mixed with 0.1% TPP (*w*/*v*) prior to cross-linking. The ionic cross-linking reaction was initiated by the slow addition of chitosan at 0.5% (*w*/*v*) under probe-type ultrasonication on ice. The photosensitizer used in this work is iron(III) phthalocyanine-4,4′,4″,4‴-tetrasulfonic acid (FePC). The fabrication procedure of FNP is illustrated in Figure 1. The concentration of FNP was determined by the content of encapsulated FePC.

### 2.3. Characterization of Nanoparticles

The fabricated nanoparticles were lyophilized and the powdered samples were placed on KBr to form pellets under compression for FT-IR measurement. The FT-IR spectra were recorded from 4000 cm^−1^ to 700 cm^−1^ at a resolution of 4 cm^−1^ with an FT-IR spectrometer (VERTEX 70v, Bruker, Billerica, MA, US) equipped with a mercury cadmium telluride detector.

The physical properties, including hydrodynamic diameter (D_h_), polydispersity indexes (PDI) and zeta-potential of fabricated nanoparticles, were measured by dynamic light scattering (DLS, Nano-ZS90, Malvern, Worcestershire, UK).

### 2.4. Determining the Encapsulation Efficiency of Chitosan/TPP Nanoparticles

Phthalocyanine-encapsulated nanoparticles were separated from the aqueous suspension containing FePC (Sigma-Aldrich, St. Louis, MO, USA) by centrifugation at 12,000 rpm for 5 min. The supernatant was collected and the free FePC was determined by measuring the absorbance at 630 nm using UV–VIS spectroscopy (S-3100, Scinco, Seoul, Korea). The encapsulation efficiency of FePC in nanoparticles was calculated as follows: [(A−B)/A] × 100, where A is the total amount of FePC and B is the amount of free FePC.

### 2.5. Determination of Release Efficiency of Phthalocyanine from Chitosan/TPP Nanoparticles

Release of FePC from nanoparticles was measured in phosphate buffer solution (PBS, Avantor, Center Valley, PA, USA) at different time intervals. Primarily, FNP was removed from free FePC by centrifugation at 12,000 rpm for 5 min. The released FePC in the supernatant was quantified by absorbance at 630 nm.

### 2.6. Culture of Planktonic C. albicans and C. tropicalis

In this study of *C. tropicalis*, *C. albicans* was used as a comparison. Both *C. tropicalis* and *C. albicans* were provided by Dalin Tzu Chi Hospital (Chia-Yi, Taiwan). Suspensions (1.5 × 10^8^ cells/mL) of *C. albicans* (ATCC 90029) or *C. tropicalis* (ATCC 13803) were cultured in yeast extract-peptone-dextrose (YEPD) growth medium in glass tubes at 37 °C. Antifungal agents or photosensitizers were added to *C. albicans* or *C. tropicalis* in YEPD growth medium and incubated at 37 °C. To determine cell viability, aliquots of 1000-fold serial dilutions of each sample were seeded on YEPD agar plates. All plates were aerobically incubated at 37 °C for 18 h, and the number of colony forming units per milliliter (CFU/mL) was counted from the plates. The tested fluconazole concentration was lower than or equal to 208 μM (64 µg/mL), the minimum inhibitory concentration (MIC) reported by Clinical Laboratory Standards Institute [38]. The tested concentrations of flucytosine were in the range of various MIC values measured from flucytosin-resistant *C. tropicalis* [39]. Notably, the activity of antifungal agents is suggested to be tested in RPMI 1640 medium [40]. In this work, we kept the *Candida* species in YEPD medium in all experiments.

### 2.7. Culture of Adherent C. tropicalis

To generate adherent cultures of *C. tropicalis*, 100 μL of a *C. tropicalis* suspension was transferred into a 96-well culture plate at a concentration of 1.5 × 10^4^ cells/mL and incubated at 37 °C in an orbital shaker at 80 rpm overnight. Subsequently, samples were washed with 150 μL of PBS to remove suspended cells. YEPD medium containing antifungal agents or photosensitizer was added for further incubation at 37 °C. The incubation with antifungal agents lasted 24 or 48 h, whereas incubation with photosensitizer lasted 4 h.

The MTT cell proliferation assay was used to measure the viability of adherent *C. tropicalis* cells. After the addition of 100 μL of medium containing 5 μL of MTT, *C. tropicalis* cells were incubated for 4 h at 37 °C in the dark, and formazan absorbance was measured at 595 nm in an ELISA reader.

A *t*-test was performed for statistical analysis in all experiments using *C. tropicalis*. The reported values are the mean values of three replicates. Statistical significance was indicated at *p* < 0.05 (*), *p* < 0.01 (**), or *p* < 0.001 (***).

### 2.8. PDT Experiments

PDT experiments were carried out by illuminating FePC- or FNP-treated *C. tropicalis* using a homemade device consisting of an array of 20 red LEDs (peak emission wavelength at 630 nm, 11.0 ± 1 mW/cm^2^). After illumination for 30 min, the accumulated photoenergy was 20 J/cm^2^. FePC or FNP solutions were added to *C. tropicalis* suspensions to reach the desired concentration. A 1 mL mixture of *C. tropicalis* and photosensitizer was transferred to a 96-well plate and incubated in the dark for 4 h. Subsequent illumination for 30 min activated the PDT. In experiments combining PDT with chemical therapy, *C. tropicalis* was treated with 128 μM flucytosine prior to or after the PDT experiments.

### 2.9. Cellular Uptake of FePC in FePC- and FNP-Treated C. tropicalis

*Candida tropicalis* was cultured in test tubes at a concentration of 1 × 10^8^ cells/mL for treatments with 80 µM FePC or FNP. After incubation for 4 h, the cells were washed twice with H_2_O and spun down at 13,000 rpm for 1 min. The cell pellet mixed with H_2_O was sonicated. Subsequently, the cell lysate was centrifuged at 13,000 rpm for 5 min. The concentration of FePC in the suspension was quantified by measuring the absorbance at 630 nm.

## 3. Results

### 3.1. Fabrication and Characterization of Nanoparticles

**Figure 2:**
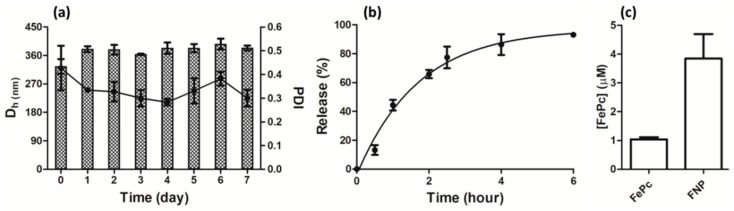
Characterization of FNP. (**a**) Time-course measurements of D_h_ and polydispersity indexes (PDI) by dynamic light scattering (DLS) shown in columns and dots, respectively; (**b**) the release of FePC from FNP; (**c**) uptake of FePC in *C. tropicalis* after treatment with 80 μM FePC or FNP for 4 h.

The chitosan/TPP nanoparticles were fabricated based on previously reported conditions [34]. The D_h_, PDI, and zeta potential of FNP measured by DLS were 325.2 ± 23.3 nm, 0.427 ± 0.094, and 26.4 ± 3.2 mV, respectively. The fabricated FNP was very stable as tested in time-course DLS measurements shown in Figure 2a, and both D_h_ and PDI were close to the initial values. The encapsulation efficiency of FePC in FNP was 80.0 ± 5.0%. In 4 h, 80% of the FePC was released from FNP into the aqueous solution, as shown in Figure 2b. We quantified FePC in *C. tropicalis* after treatment with FePC or FNP. As shown in Figure 2c, the uptake of FePC was very limited. Significantly, the uptake of FePC was enhanced four-fold when treated with FNP.

### 3.2. Spectroscopic Characterization of Nanoparticles

**Figure 3:**
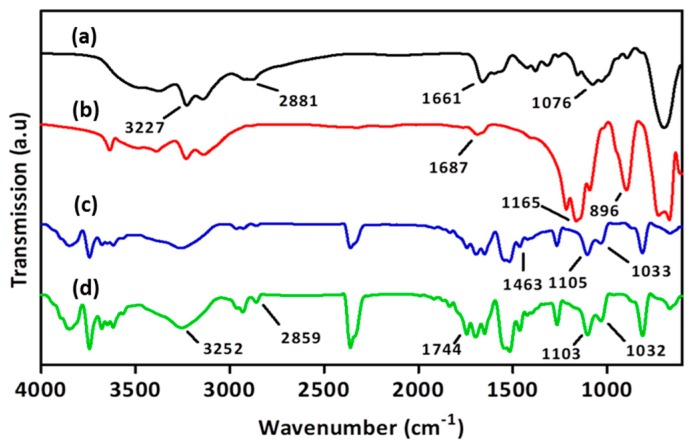
FT-IR spectra of (**a**) chitosan; (**b**) TPP; (**c**) FePC; and (**d**) FNP.

The composition of nanoparticles was confirmed by FT-IR for the vibrational features of the functional groups. As shown in Figure 3a, the FT-IR spectrum of chitosan reveals stretches of O–H and N–H at 3200–3600 cm^−1^, C=O at 1661 cm^−1^, C–O at 1076 cm^−1^, and C–H (sp^3^) at 2800–3000 cm^−1^. TPP can be recognized by FT-IR bands at 1165 and 896 cm^−1^ for P=O and P–O–P stretching, respectively, as shown in Figure 3b. The FT-IR spectrum of FePC illustrated in Figure 3c includes C=N stretching of pyrrole at 1463 cm^−1^, Fe–N of pyrrole at 1105 cm^−1^ and –SO_3_ stretching at 1033 cm^−1^ [41]. In FNP, vibrational bands representing chitosan, TPP, and FePC are all present, as shown in Figure 3d.

### 3.3. Viability of C. tropicalis after Treatment with Antifungal Agents

**Figure 4:**
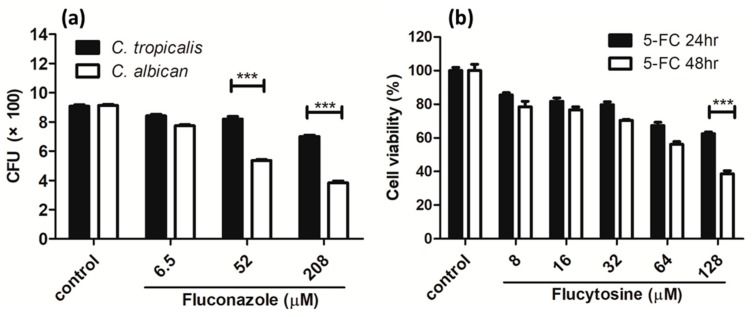
Viability of *C. tropicalis* after treatment with (**a**) fluconazole in planktonic culture in comparison to *C. albican* and (**b**) flucytosine (5-FC) in adherent culture. *** *p* < 0.001.

In order to test whether the *C. tropicalis* used in the experiment is resistant to fluconazole, the mostly commonly used antifungal medicine, the antifungal effect of fluconazole in the *Candida* species was carried out. The fluconazole concentration was 208 μM or less. A non-drug resistant strain of *C. albican* was tested as a comparison. As shown in Figure 4a, dose-dependent inhibition was very pronounced in *C. albicans*. Comparatively, dose-dependence was very weak for *C. tropicalis*. Importantly, the inhibition of *C. tropicalis* was a lot weaker than the inhibition of *C. albicans*, especially at 208 μM. This indicates that *C. tropicalis* is strongly resistant to fluconazole.

As *C. tropicalis* has a strong resistance to fluconazole, other antifungal medicines are used in the clinic. Flucytosine, a well-known azole antifungal medicine, is one of the commonly used medications for *C. tropicalis* infections. Therefore, we tested the resistance of fluconazole-resistant *C. tropicalis* to flucytosine. The flucytosine concentration was 128 μM or less. As shown in Figure 4b, a weak dose-dependence was shown after 24 h of treatment. The dose-dependence was slightly stronger in the 48 h-treatment experiment. However, after the 24 h treatment, more than half of *C. tropicalis* survived at the maximum concentration of flucytosine. The cell viability was ~40% after the 48 h-treatment. This viability assay indicates that *C. tropicalis* is resistant to flucytosine.

### 3.4. PDT Effect on Planktonic and Adherent C. tropicalis

**Figure 5:**
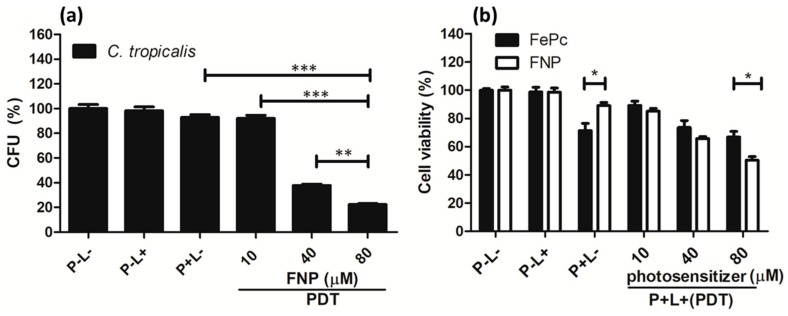
Viability of *C. tropicalis* in (**a**) planktonic cultures with FNP-PDT, and (**b**) adherent cultures after PDT with FePC or FNP. P and L represent photosensitizer and light, respectively. The symbol + indicates that the specific factor was used, and – indicates that the specific factor was not used. * *p* < 0.05, ** *p* < 0.01, or *** *p* < 0.001.

Antimicrobial PDT using FNP against *C. tropicalis* was performed in both planktonic and adherent cultures. As shown in Figure 5a, in planktonic cultures, PDT with 40 and 80 μM of FNP caused significant inhibition. This antimicrobial effect was much better than the effect achieved with antifungal medicines, such as fluconazole and flucytosine, which are shown in Figure 4.

*Candida* species which develop biofilms are more drug-resistant and virulent [42]. *Candida* species develop biofilms when they are dispersed. Therefore, antimicrobial PDT using FNP was performed in adherent cultures of *C. tropicalis*. As shown in Figure 5b, cell viability decreased after performing PDT with FePC or FNP. The antimicrobial PDT effect was better when using FNP as compared to FePC. However, the antimicrobial PDT effect in adherent *C. tropicalis* was a lot weaker than in planktonic *C. tropicalis*. Consistent with our previous study on *C. albicans* [25], this result fulfills our prediction that it is more difficult to eradicate *C. tropicalis* when it is forming biofilms.

### 3.5. The Effect of Combined Therapy using Flucytosine and FNP-PDT on C. albicans in Adherent Cultures

**Figure 6:**
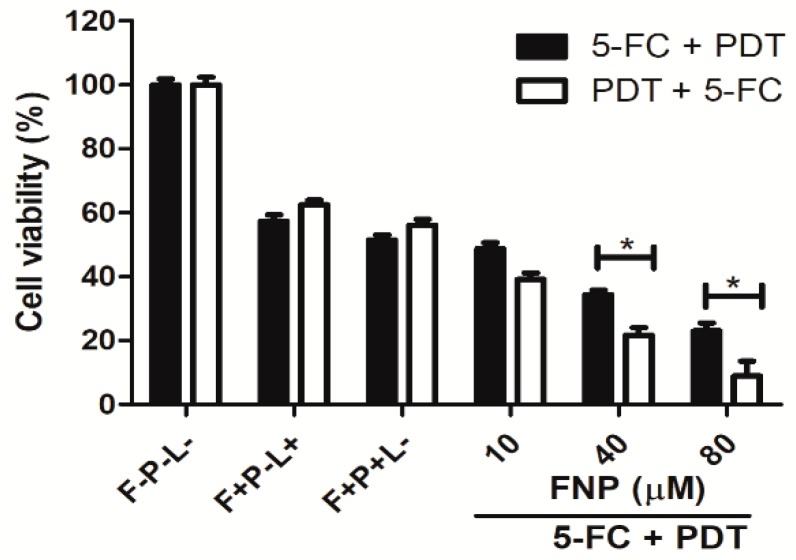
Viability of *C. tropicalis* in the combined therapy of flucytosine (5-FC) and FNP-PDT sequentially shown in the solid columns, and the reversed order shown in the unfilled columns. F, P and L represent flucytosine, photosensitizer and light, respectively. The symbol + indicates that the specific factor was used, and – indicates that the specific factor was not used. *p < 0.05.

To fully eradicate *C. tropicalis*, we combined traditional flucytosine therapy and FNP-PDT. The antimicrobial effect of this combination was also tested in different sequences. As shown in Figure 4, flucytosine (128 μM) caused 40–60% cell death. As shown in Figure 6, the photosensitizer (FNP) had no effect on cell viability before illumination. After the flucytosine treatment, FNP-PDT largely decreased the viability of *C. tropicalis* cells in a dose-dependent manner. Cell viability was ~25% when the treatment involved flucytosine and subsequent PDT with 80 μM FNP. Alternatively, using FNP-PDT prior to the flucytosine therapy caused more significant cell death. The cell viability was only ~10% when PDT was used with 80 μM FNP and flucytosine treatment sequentially.

### 3.6. Cellular Morphology of C. tropicalis

**Figure 7:**
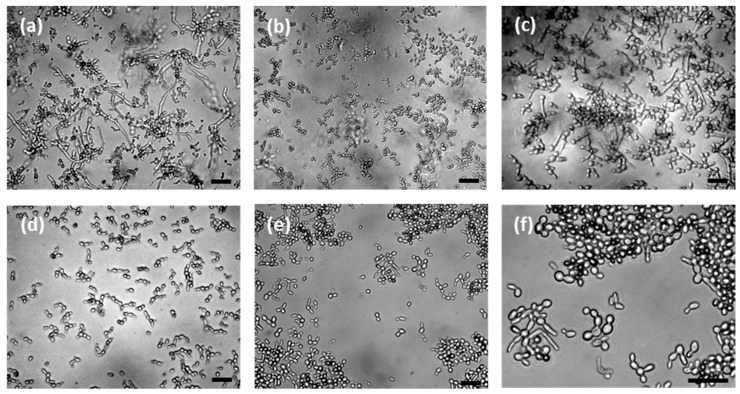
The morphology of *C. tropicalis* (**a**) before the treatment, and after treatment with (**b**) FNP-PDT; (**c**) flucytosine, and combination treatments of (**d**) flucytosine for 24 h followed by FNP-PDT, and (**e**) FNP-PDT followed by flucytosine treatment for 24 h; (**f**) An enlarged image of (e). The scale bar represents 10 μm.

A unique feature of adherent *C. tropicalis* cells was their long pseudohyphae, as shown in Figure 7a. After FNP-PDT, the long pseudohyphae were greatly eradicated, as shown in Figure 7b. In contrast, flucytosine was toxic to yeast-like *C. tropicalis*. Flucytosine shortened the pseudohyphae but could not eliminate them, and the pseudohyphae were still widely present in Figure 7c. After the sequential treatment of flucytosine and FNP-PDT, as shown in Figure 7d, very few *C. tropicalis* cells were present, and the pseudohyphae were greatly eliminated. The eradication of pseudohyphae was more complete when FNP-PDT was performed prior to flucytosine treatment, as shown in Figure 7e,f. These images illustrate mostly yeast-like oval cells rather than long pseudohyphae. It is very clear that the *C. tropicalis* pseudohyphae and yeast-like cells were eradicated completely in this sequential FNP-PDT and flucytosine treatment.

## 4. Discussion

Infection of *C. tropicalis* has become an important issue, especially for the strains resistant to traditional antifungal agents. The strain of *C. tropicalis* used in this study is resistant to fluconazole and flucytosine. Clinically, it is very difficult to treat infections caused by this strain. Therefore, alternative antimicrobial therapies are needed. As an alternative treatment, PDT is an efficient antitumor therapy. However, for antimicrobial therapies, the effect of PDT is limited, as has been previously reported [25,43], and this is confirmed in this work. One major problem in antimicrobial PDT is the poor solubility and limited cell penetration of photosensitizers [44]. In this work, we encapsulated FePC with cationic chitosan/TPP nanoparticles. This approach solved the problem of solubility and provided a targeting effect through the charge interaction between the cationic chitosan/TPP nanoparticles and the anionic surface on the cell walls [45]. This encapsulation enhanced four-fold the availability of FePC to *C. tropicalis* cells. However, after the uptake enhancement using cationic chitosan/TPP nanoparticles, the uptake of FePC was only 5%. This result strongly emphasized the importance of the drug delivery to microbes. How to greatly improve the cellular availability of therapeutics is a critical issue for all antimicrobial therapies.

In our study, FNP-PDT inhibited planktonic *C. tropicalis* very efficiently. However, FNP-PDT was not highly toxic against adherent *C. tropicalis*. As judged from the morphology, FNP-PDT abolished most pseudohyphae rather than killing the *C. tropicalis* cells. This finding on the effect of PDT on biofilms is consistent with previous studies on *Candida* species [25,46,47] and *Staphylococcus aureus* [48]. In contrast to PDT, as an antifungal agent, flucytosine killed the *C. tropicalis* cells but could not eliminate the pseudohyphae efficiently. As the antibiotic resistance is mainly ascribed to the secretion of the extracellular matrix from hyphal and pseudohyphal organisms in biofilms [49], PDT and an antifungal treatment can be complementary. As proved in this study, the combined use of FNP-PDT and flucytosine greatly inhibits *C. tropicalis* replication. Interestingly, the sequence of these two treatments was critical to the viability of *C. tropicalis*. The use of FNP-PDT prior to flucytosine treatment provided better inhibition than the reverse sequence. The antifungal treatment of candidiasis usually takes months [50]. During the hours of flucytosine treatment, surviving *C. tropicalis* continued to develop pseudohyphae, weakening the antifungal effect. When the pseudohyphae were omnipresent, the subsequent FNP-PDT could only eliminate part of the pseudohyphae, resulting in the survival of *C. tropicalis* with long pseudohyphae. Therefore, the most efficient approach is to eradicate pseudohyphae by FNP-PDT prior to the treatment with antifungal agents to toxify *C. tropicalis* cells.

The resistance to antimicrobial agents is the most serious issue in antimicrobial therapy. After finding the great effect of PDT on biofilms of *Candida* species and bacteria, we need to further examine whether the PDT-treated strains overproduce drug-efflux proteins to remove photosensitizers.

## 5. Conclusions

The antibiotic resistance of *Candida* species is an important issue, and the development of alternative therapies to kill drug-resistant *Candida* cells and to reduce their biofilm-associated virulence is needed. Antifungal agents greatly eliminate the yeast form of *C. tropicalis*, but have little effect on pseudohyphae. Enhanced antimicrobial PDT using FePC-encapsulated chitosan/TPP nanoparticles can effectively eradicate pseudohyphae. The sequential use of FNP-PDT and flucytosine can strongly eliminate pseudohyphae, to reduce the virulence and inhibit the growth of fluconazole-resistant *C. tropicalis* cells. Potentially, this strategy can be applied to other drug-resistant pathogens.
